# Seasonal Childhood Anaemia in West Africa Is Associated with the Haptoglobin 2-2 Genotype

**DOI:** 10.1371/journal.pmed.0030172

**Published:** 2006-05-02

**Authors:** Sarah H Atkinson, Kirk Rockett, Giorgio Sirugo, Philip A Bejon, Anthony Fulford, Maria A O'Connell, Robin Bailey, Dominic P Kwiatkowski, Andrew M Prentice

**Affiliations:** **1**Medical Research Council Laboratories, Banjul, The Gambia; **2**Medical Research Council International Nutrition Group, Nutrition and Public Health Intervention Research Unit, London School of Hygiene and Tropical Medicine, London, United Kingdom; **3**Wellcome Trust Centre for Human Genetics, University of Oxford, Oxford, United Kingdom; **4**Medical Research Council, Human Nutrition Research, Cambridge, United Kingdom; Royal Melbourne HospitalAustralia

## Abstract

**Background:**

Anaemia is a major cause of morbidity and mortality for children in Africa. The plasma protein haptoglobin (Hp) binds avidly to free haemoglobin released following malaria-induced haemolysis. Haptoglobin polymorphisms result in proteins with altered haemoglobin-binding capacity and different antioxidant, iron-recycling, and immune functions. Previous studies examined the importance of haptoglobin polymorphism in malaria and iron homeostasis, but it is unknown whether haptoglobin genotype might be a risk factor for anaemia in children in a malaria-endemic area.

**Methods and Findings:**

A cohort of 780 rural Gambian children aged 2–6 y was surveyed at the start and end of the malaria season. Samples were taken to assess haemoglobin (Hb) concentration, iron status (ferritin, zinc protoporphyrin, transferrin saturation, and soluble transferrin receptor concentration), haptoglobin concentration, α-1-antichymotrypsin (a measure of inflammation), and malaria parasites on blood film. We extracted DNA and genotyped for haptoglobin, sickle cell, and glucose-6-phosphate (G6PD) deficiency. Mean Hb levels fell over the malaria season. Children with the haptoglobin 2-2 genotype (17%) had a greater mean drop in Hb level over the malaria season (an 8.9 g/l drop; confidence interval [CI] 5.7, 12.1) compared to other children (a 5.1 g/l drop; CI 3.8, 6.4). In multivariate regression analysis, controlling for baseline Hb level, age group, village, malaria parasites on blood film, iron status, haptoglobin concentration, and G6PD deficiency, haptoglobin genotype predicted Hb level at the end of the malaria season ( *p* = 0.0009, coefficient = −4.2). Iron status was not influenced by haptoglobin genotype.

**Conclusions:**

The finding that haptoglobin 2-2 genotype is a risk factor for anaemia in children in a malaria-endemic area may reflect the reduced ability of the Hp2-2 polymer to scavenge free haemoglobin-iron following malaria-induced haemolysis. The magnitude of the effect of haptoglobin genotype (4 g/l Hb difference, *p* = 0.0009) was comparable to that of G6PD deficiency or HbAS (3 g/l difference, *p* = 0.03; and 2 g/l difference, *p* = 0.68, respectively).

## Abstract 2

Haptoglobin 2-2 genotype seems to be a risk factor for anaemia in children in a malaria-endemic area, possibly reflecting the reduced ability of the Hp2-2 polymer to scavenge free haemoglobin-iron following malaria-induced haemolysis.

## Introduction

Anaemia (haemoglobin [Hb] < 110 g/l) is a serious public health problem affecting more than half of children less than 5 y of age in malaria-endemic countries of Africa; in a survey in The Gambia 76% of children were anaemic [ 1]. Anaemia has multiple causes, including malaria and micronutrient deficiencies [ 2]. In sub-Saharan Africa 20% to 40% of children have undetectable levels of haptoglobin (Hp) due to haemolysis, and low levels of haptoglobin are strongly associated with malaria infection [ 3]. Hp, an acute-phase plasma protein, is characterised by its strong binding affinity (>10 ^10^ mol ^−1^) for free haemoglobin released following haemolysis. The *Hp ^1^* and *Hp ^2^* alleles are encoded by a single gene on Chromosome 16; the *Hp ^2^* allele was formed from an intragenic duplication originating from a nonhomologous crossing-over of two *Hp ^1^* alleles [ 4]. Haptoglobin exists in three common phenotypes: the homodimer Hp1-1, the linear polymer Hp1-2, and the large circular polymer Hp2-2 [ 5]. Clear functional differences exist between the phenotypes, including differences in modulation of oxidant stress, recycling of haem-iron, and immune function [ 5]. Haemolytic stress is likely to accentuate differences between the phenotypes, as has been found in haptoglobin knock-out mice compared to wild-type mice [ 6].

The Hp2-2 polymer has very different biochemical and biophysical properties compared to Hp1-1 and Hp1-2 [ 5]. The Hp2-2 protein is present in lower concentrations [ 7] and binds less efficiently to free haemoglobin; Hp1-1 and Hp1-2 have higher binding affinities [ 8]. These differences are reflected in vivo by altered oxidant defence and iron handling. Vitamin C concentrations were significantly lower in the Hp2-2 phenotype (49.9 μmol/l), but did not differ between Hp1-1 and Hp1-2 individuals (61.5 μmol/l and 63.7 μmol/l respectively) [ 9]. Furthermore, in Hp2-2 individuals iron is delocalised into poorly exchangeable storage compartments of the mononuclear phagocytic system; excess monocyte iron was found in the Hp2-2 phenotype (687 μg/g L-ferritin) compared to the Hp1-1 and Hp1-2 phenotypes (326 μg/g and 366 μg/g L-ferritin, respectively) [ 10]. However, it is not known whether Hp2-2 phenotype might be a risk factor for anaemia in an environment of malaria-induced haemolysis and limited dietary iron availability.

The pathogenesis of malarial anaemia is complex and includes haemolysis, accelerated erythrophagocytosis, and cytokine-induced dyserythropoiesis [ 11, 12]; reduced antioxidant defence [ 13, 14] and a shift in iron distribution from functional to storage compartments [ 15, 16] have also been found. The association between haptoglobin and susceptibility to malaria infection is controversial, and published reports are conflicting. The Hp2-2 phenotype was associated with protection from severe malaria and placental infection in a number of case control studies [ 17– 20]. However, a large study analyzing haptoglobin genotypes and a recent case control study did not find an association [ 21, 22].

We hypothesised that the Hp2-2 phenotype, due to known functional differences, may be a risk factor for anaemia in children in an environment of malaria and relative iron deficiency. We thus investigated the possibility that children homozygous for the *Hp ^2^* allele would have a lower haemoglobin level after the malaria season. To control for the multifactorial aetiology of anaemia we measured baseline haemoglobin levels in the same children prior to the malaria season, and other factors that might influence haemoglobin level. We explored the interaction with plasma iron markers and compared the impact of haptoglobin with other genetic polymorphisms that might influence haemoglobin levels over the malaria season; namely, HbS and the G6PD A and A− variants common to sub-Saharan Africa [ 23].

## Methods

### Patients and Methods

Ethical permission for the study was granted by the Gambian Government and Medical Research Council Ethics Committee, and Gambian National DNA Collection Guidelines were followed regarding the handling of genetic material and information. Parental written informed consent was obtained for all study participants.

**Figure 1:**
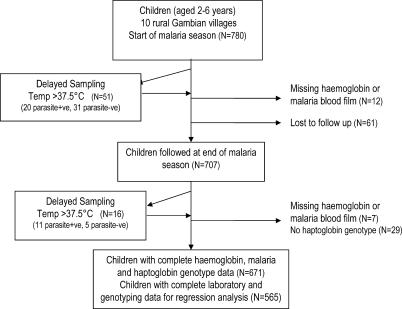
Sample Construction At the start of the malaria season, 780 children (aged 2–6 y) were recruited from ten rural Gambian villages, of these, 61 children were lost to follow-up (of whom four died). At the end of the malaria season, 707 children were surveyed. A total of 671 children had complete haemoglobin, malaria blood film, and haptoglobin genotype data. After biochemical assays and further genotyping, 565 children had complete data for multivariate regression analysis.

A cohort of 780 children aged from 2 to 6 y was recruited from ten rural villages in the West Kiang region of The Gambia at the start of the malaria season, July 2001, with follow-up to December 2001/January 2002. All children were eligible except those with serious chronic illness or those enrolled in another study. Figure 1 provides an overview of the study population. Ethnic groups were Mandinka (nine villages, 700 children) and Fulani (one village, 80 children). Children had anthropometric measurements taken and were examined by the study clinician. All children received a 3-d course of mebendazole at the start of the study for possible hookworm infection. A blood sample was collected for full blood count, malaria slide, iron status assays, haptoglobin concentration, α-1-antichymotrypsin (a marker of inflammation), and DNA extraction. Children with a temperature over 37.5 °C had a malaria blood film, appropriate clinical treatment, and a blood sample 2 wk later after recovery from illness. Children with malaria parasites on blood film were treated with chloroquine and pyrimethamine-sulfadoxine (Fansidar) according to Gambian Government guidelines. This procedure was repeated at the end of the malaria season for each child.

Malaria incidence is highly seasonal in The Gambia with the majority of malaria cases occurring between September and December [ 24]. Haemoglobin levels in children from the study area were previously found to be highest in July and lowest in November [ 25]. We thus sampled at the start and end of the malaria season to assess the effect of haptoglobin genotype on haemoglobin levels in the malaria season compared to baseline levels. Children were followed across the malaria season to control for multiple individual factors that may influence haemoglobin levels.

### Laboratory Procedures and Statistical Analysis

Haemoglobin level was measured by the Medonic CA 530 Oden 16 Parameter System Haemoglobinometer and zinc protoporphyrin level by the Aviv Biomedical Haematoflurometer, within 24 h of collection; daily quality controls were performed against commercial standards, and duplicate samples were run. Blood films were stained with Giemsa and examined for malaria parasites according to standard methods. Frozen plasma samples were analysed at MRC Human Nutrition Research Laboratories, Cambridge. Haptoglobin concentration, a marker of recent haemolysis, was measured by immunoturbidimetry, Tina-quant on the Cobas Bioanalyser (Roche Systems, Basel, Switzerland). We measured ferritin concentration by the Imx Ferritin assay, a Microparticle Enzyme Immunoassay (MEIA), (Abbott Laboratories, Abbott Park, Illinois, United States) and soluble transferrin receptor (sTfR) concentration by enzyme-linked immunosorbent assay (ELISA) using a commercially available kit (R&D Systems, Minneapolis, United States). Serum iron and unsaturated iron binding capacity (UIBC) were measured by Ferrozine-based photometry and colorimetry using an automated analyser (Hitachi 911, Hitachi, Tokyo, Japan). Transferrin saturation (TS, %) was calculated from plasma iron and unsaturated iron binding capacity (TS = [plasma iron/(UIBC + plasma iron)] × 100). We also measured α _1_-antichymotrypsin, a measure of the inflammatory response, by immunoturbidimetry (Cobas Mira Plus Bioanalyser), to aid the interpretation of the markers of iron status.

Genotyping (as opposed to the commonly used electrophoretic phenotyping) allowed haptoglobin typing of patients regardless of possible hypohaptoglobinaemia secondary to subclinical malaria. DNA was extracted from peripheral blood leucocytes according to standard methods [ 26] and quantified using the PicoGreen assay with measurement of fluorescence by the TECAN SPECTRAfluor Plus fluorimeter. Haptoglobin was genotyped by allele-specific PCR, according to a method modified from Yano et al. [ 27]. The products were resolved in 1.2% agarose gel, stained with ethidium bromide, and visualized under UV light. The oligonucleotide sequences of the haptoglobin primers and the PCR conditions are detailed in Tables S1 and S2, respectively. HbS and G6PD deficiency (A, A−) polymorphisms were genotyped on amplified DNA (Primer Extension Pre-amplification) [ 28] by typing single nucleotide polymorphisms with Sequenom technology using MALDI-TOF mass spectrometry. The oligonucleotide sequences of the primers for HbS and the G6PD A and A− type deficiency polymorphisms are in Table S3.

### Statistical Analysis

Analyses were conducted using S TATA version 8.0 (Stata, Timberlake, London, United Kingdom). Pearson's Chi-square test assessed associations between haptoglobin genotype and other genetic polymorphisms, and binomial regression was used to test for differences in allele frequency between villages. Normality diagnostics were performed and continuous variables that were not normally distributed were log-transformed. Weight for height Z-scores were calculated using Epi Info 2000 software. Prior functional data suggested the Hp2-2 phenotype had unique properties compared to the Hp1-2 or Hp1-1 phenotypes [ 9, 10]. This study was designed to test the hypothesis that these functional differences would cause in vivo differences in iron and haemoglobin handling after malaria-induced haemolysis. Thus the primary analysis compares Hp2-2 with the Hp1-2 and Hp1-1 groups. A secondary analysis treated each genotype separately.

The effect of haptoglobin and other genotypes on the drop in haemoglobin was first assessed by Student's t-test. Further analysis, using stepwise multivariate linear regression, controlled for other variables likely to influence haemoglobin level at the end of the malaria season. Here, rather than normalising haemoglobin values by calculating the drop during the malaria season, baseline haemoglobin value was used as an explanatory variable in the model. Age group, malaria parasites (on blood film), village, and baseline haemoglobin level were first included in the model. Other variables were added manually (in order: sex, markers of iron status [zinc protoporphyrin, ferritin, soluble transferrin receptor and transferrin saturation], weight for height Z-score, α _1_-antichymotrypsin, haptoglobin concentration, G6PD deficiency, and HbS) and retained if *p* < 0.1. Haptoglobin genotype was added last. The validity of the assumptions of normality and constant variance were confirmed with residual plots.

## Results

### Characteristics of Study Population

Haptoglobin genotypes were in Hardy-Weinberg equilibrium. Figure 1 shows numbers of children lost to follow-up and those with pyrexia who were sampled 2 wk later following clinical management; haptoglobin genotype distribution did not differ in either of these groups of children. Four children died; cause of death was severe malarial anaemia in two (one of whom had sickle cell disease), cerebral malaria in one, and the cause was unknown in the fourth. Binomial regression analysis did not reveal significant geographic clustering of the *Hp ^2^* allele by village, and no association was found between haptoglobin genotype and either sickle genotype or G6PD deficiency. Table 1 summarises the characteristics of the sample by haptoglobin genotype.

**Table 1:**
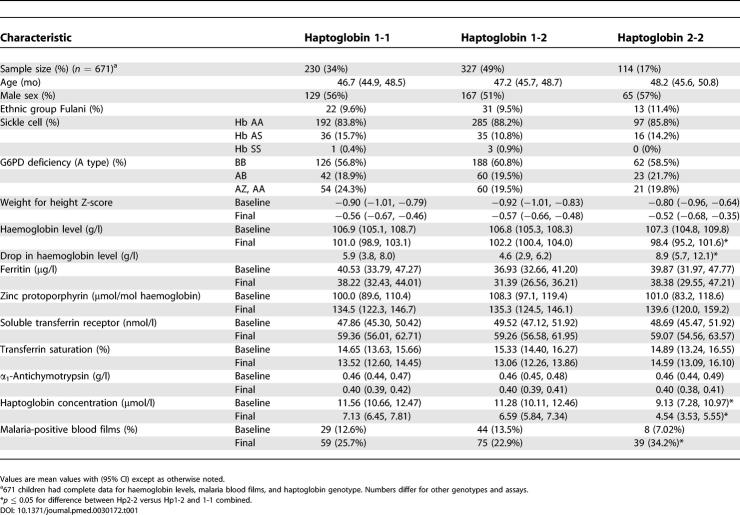
Characteristics of Study Population by Haptoglobin Genotype

### Univariate Analysis

**Figure 2:**
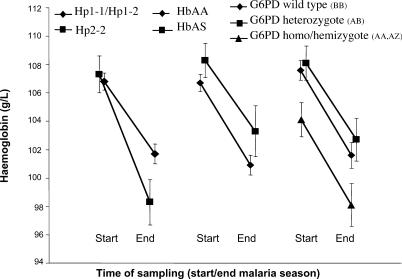
Drop in Haemoglobin Level during the Malaria Season according to HbAS, G6PD Deficiency, and Haptoglobin Genotype Haemoglobin levels at the start and end of the malaria season are shown by haptoglobin genotype ( *Hp ^2/2^* versus *Hp ^1/1^* and *Hp ^1/2^* combined), HbAS compared to HbAA and G6PD (A type) deficiency, wild-type, heterozygotes, and homozygotes/hemizygotes. Error bars denote standard error of the mean.

Overall, mean haemoglobin levels fell across the malaria season; from 106.9 g/l (95% confidence interval [CI _95%_] 105.9,108.0) to 101.2 g/l (CI _95%_ 99.9,102.4) by the end of the malaria season; a mean drop of 5.8 g/l (CI _95%_ 4.5,7.0) ( *p* = 0.0001). In primary analysis, children carrying the *Hp ^2/2^* genotype had a mean drop in haemoglobin of 8.9 g/l (CI _95%_ 5.7,12.1) compared with 5.1 g/l (CI _95%_ 3.8,6.4) for the other genotypes ( *p* = 0.02 by Student's t-test). The drop in haemoglobin was similar for the *Hp ^1/1^* and *Hp ^1/2^* genotypes (5.9 g/l and 4.6 g/l respectively). In secondary analysis an ANOVA for the three genotypes also indicated a greater drop in mean haemoglobin level in children with the *Hp ^2/2^* genotype ( *p* = 0.045). By comparison, sickle genotype and G6PD deficiency did not significantly influence drop in haemoglobin level over the malaria season. Drop in haemoglobin level over the malaria season for haptoglobin, HbAS, and G6PD A type deficiency is shown in Figure 2. Baseline haemoglobin levels did not differ in the haptoglobin genotypes, but were lower by the end of the malaria season in children carrying the *Hp ^2/2^* genotype ( *p* = 0.05 in univariate analysis, *Hp ^2/2^* versus *Hp ^1/2^* and *Hp ^1/1^* combined). As found previously [ 7], children carrying the *Hp ^2/2^* genotype had lower mean haptoglobin concentrations. We also found an increased number of asymptomatic children with malaria parasitaemia in the *Hp ^2/2^* group at the end of the malaria season ( *p* = 0.02 for *Hp ^2/2^* versus *Hp ^1/1^* and *Hp ^1/2^* combined, *p* = 0.06 for heterogeneity among the three genotypes).

### Multivariate Regression Analysis

Multiple factors influenced haemoglobin level. A multivariate regression analysis was performed with haemoglobin level at the end of the malaria season as dependent variable. In this model, baseline haemoglobin level, village, age group, malaria parasites on blood film, iron status, haptoglobin concentration (a measure of haemolysis in the last 10 d), G6PD A type deficiency, and haptoglobin genotype ( *p* = 0.0009, coefficient = −4.2) emerged as significant predictors of haemoglobin level at the end of the malaria season ( Table 2). Sex, baseline iron markers (log ZnPP and log soluble transferrin receptor), weight for height Z-scores, α _1_-antichymotrypsin levels, and sickle genotype were not retained in the model, as the *p*-value exceeded 0.1. In a secondary analysis, *p* = 0.002 for heterogeneity among the three haptoglobin genotypes. The *Hp ^1/1^* and *Hp ^1/2^* genotypes did not differ (1 g/l difference, *p* = 0.32). A further multivariate analysis with change in Hb as the dependent variable found a similar effect for haptoglobin genotype ( *p* = 0.007, coefficient = −4.1). Iron status (as measured by ferritin, zinc protoporphyrin, transferrin saturation, and soluble transferrin receptor concentration) strongly influenced haemoglobin level, but was not significantly altered by haptoglobin genotype.

**Table 2:**
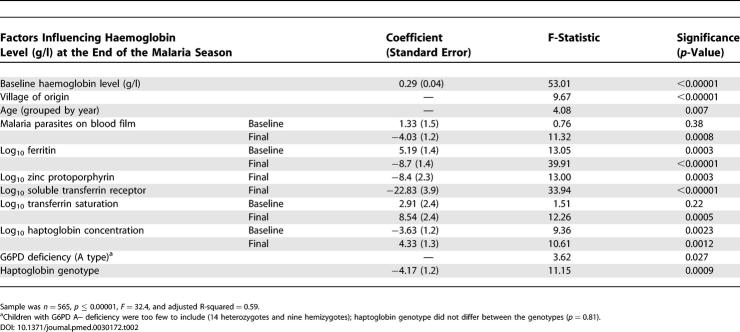
Multivariate Regression Analysis for Haemoglobin Level at the End of the Malaria Season

## Discussion

Multiple factors contributed to the complex aetiology of anaemia in young rural Gambian children; both iron status and malaria infection emerged as strong predictors of haemoglobin level. We speculated that haptoglobin, encoded by a gene that regulates haemoglobin-iron metabolism after haemolysis, might critically influence haemoglobin levels in an environment of malaria-induced haemolytic stress and iron deficiency. Haemoglobin levels fell generally over the malaria season, but this effect was concentrated in children carrying the *Hp ^2/2^* genotype (who had approximately twice the drop in Hb of children carrying the *Hp ^1/2^* genotype). Baseline haemoglobin levels were similar by Hp genotype, but by the end of the malaria season differed significantly in the *Hp ^2/2^* genotype (17% of children) compared to the *Hp ^1/1^* and *Hp ^1/2^* genotypes (coefficient = −4.2, *p* = 0.0009 in a multivariate regression analysis). The effect of *Hp ^2/2^* was greater than that of previously described genetic variants such as G6PD A type deficiency (3 g/l difference, *p* = 0.03) and HbAS (2 g/l difference, *p* = 0.68). The difference by haptoglobin genotype was robust to adjustments for other factors influencing haemoglobin level and is also supported by our retrospective analysis of a cross-sectional study of haptoglobin phenotypes conducted in this population in the 1970s [ 29]. This found that the Hp2-2 phenotype was associated with haemoglobin levels of less than 120 g/l (odds ratio = 1.92, *p* = 0.028, *n* = 825). A study from Melanesia has also reported similar differences in the 0- to 4-y age group in a malaria-endemic area (albeit based on a small sample size, *n* = 41) [ 30].

So how might the *Hp ^2/2^* genotype result in anaemia under conditions of malaria-induced haemolysis and iron deficiency? We propose three possible explanations for impaired haematological recovery from malaria infection in the *Hp ^2/2^* genotype. These mechanisms need not be mutually exclusive.

Firstly, impaired iron recycling in the *Hp ^2/2^* genotype due to delocalisation of iron into poorly exchangeable macrophage storage compartments may result in iron-deficient erythropoiesis. The haemoglobin-scavenging macrophage receptor CD163 has greater functional affinity for the Hp2-2-haemoglobin complex compared to the Hp1-1-Hb complex [ 31] resulting in significantly increased monocyte iron trapping in Hp2-2 patients [ 10]. We therefore hypothesized that prolonged or recurrent episodes of malaria would result in less efficient recycling of haemoglobin iron and iron-deficient erythropoiesis. However, we did not find an association between haptoglobin genotype and markers of iron status; it is possible, though, that monocyte iron levels may have differed between the genotypes. The association between iron homeostasis and haptoglobin genotype is controversial; some studies have found that the *Hp ^2/2^* genotype is associated with iron loading [ 10, 32], and others have not found an association [ 33– 35]. Further studies are in progress to assess the associations between haptoglobin genotype, monocyte-iron levels, and malaria infection.

Secondly, the *Hp ^2/2^* genotype may influence haematological status by failing to quench haemoglobin-iron-mediated oxidant stress. In vitro work found that the Hp 2-2 protein was associated with reduced inhibition of oxidation of low-density lipoprotein (LDL), increased redox active iron and increased oxidant stress levels compared to the Hp1-1 protein [ 36, 37]. These effects have been confirmed in vivo; Hp2-2 individuals have reduced vitamin C levels [ 9, 38], reduced ferroxidase activity if smokers [ 39], and significantly higher oxLDL/LDL ratios if male [ 40]. Malarial anaemia is associated with a reduction in red cell membrane antioxidants [ 13] and increased markers of oxidant stress [ 41]. Moreover, evidence suggests that oxidant damage to the red cell membrane leads to accelerated erythrophagocytosis via aggregation of band 3 proteins and binding of autologous IgG and complement [ 42, 43].

A third possibility is modulation of inflammatory cytokine balance. Haptoglobin promotes Th1 over Th2 activation in mice experiments [ 44] and suppresses monocyte production of tumour necrosis factor-α, IL-10, and IL-12 in response to lipopolysaccharide [ 45]. It is speculated that cross-linking of the macrophage CD163 receptor by the multimeric Hp2-2-haemoglobin complex mimics antibody binding and triggers a signalling cascade resulting in increased secretion of anti-inflammatory cytokines [ 31, 46]. In support of this idea, cross-linking of the CD163 receptor by haptoglobin-haemoglobin complexes was found to strongly stimulate IL-10 secretion in cultured macrophages [ 47]. Additionally, macrophage iron loading, as might be seen in the *Hp ^2/2^* genotype, is also associated with a reduced respiratory burst and decreased nitric oxide, IFNγ, and TNF production [ 48]. The significance of these possible effects remains to be clearly elucidated but it is possible that by dampening the inflammatory response Hp2–2 individuals may increase the chronicity of malaria infections.

The *Hp ^2^* allele is thought to have spread under strong genetic pressure, and haptoglobin disease associations are reviewed in Langlois and Delanghe (1996) [ 5]. The strongest disease pressure in this age group in rural Africa would be from malaria. Malaria infects more than 50 million children each year, and in many parts of Africa the average child has several malaria infections a year and parasites in the blood almost continuously [ 49]. Furthermore, the *Hp ^2/2^* genotype, present in 17% of children in our study, is also common. Whether there is an association between haptoglobin polymorphisms and susceptibility to malaria infection remains controversial; studies indicate either a protective or neutral effect of *Hp ^2/2^* genotype [ 17– 22]. We found an increase in asymptomatic malaria parasitaemia in the *Hp ^2/2^* genotype at the end of the malaria season ( *p* = 0.02 for *Hp ^2/2^* versus *Hp ^1/2^* and *Hp ^1/1^* combined). A further possibility is that the *Hp ^2/2^* genotype might be associated with asymptomatic, chronic parasitaemia that contributes to anaemia, but may also provide protection against severe malaria.

In summary, our results suggest that the *Hp ^2/2^* genotype is a risk factor for childhood anaemia in malaria-endemic countries. The high prevalence of a potentially detrimental allele might be explained by balancing selection pressures. An intriguing possibility is that the *Hp ^2/2^* genotype may protect against life-threatening malaria [ 18, 19] at the expense of impaired haematological recovery from mild and asymptomatic malaria. The effect of haptoglobin genotype in other haemolytic diseases, such as sickle cell, is yet to be elucidated. Further research is underway to reconfirm this finding and to investigate the potential mechanisms of anaemia.

## Supporting Information

Table S1 Oligonucleotide Sequences of the Haptoglobin Primers (23 KB DOC)

Table S2 Allele-Specific Polymerase Chain Reaction Conditions (38 KB DOC)

Table S3 Oligonucleotide Sequences of First Round and Extension Primers Designed by Sequenom Primer Designer Software for G6PD (A and A−) and HbS (33 KB DOC)

### Accession Numbers

The Vega Nucleotide Sequence Database ( http://vega.sanger.ac.uk/index.html) gene identification numbers are OTTHUMG00000073117 for HP and OTTHUMG00000024237 for G6PD. The dbSNP (build 125) reference number for the polymorphism for sickle-cell anaemia (HbS) is rs334.

**Patient Summary**

**Background.**

Anaemia is very common in African children. There are many different causes of the anaemia, including infections with malaria, worms, and other parasites. Anaemia is particularly important in children, as it can affect how well they participate at school, as well as how they recover from other illnesses.

**Why Was This Study Done?**

There is a protein called haptoglobin whose job it is to pick up and help remove from the body the haemoglobin that is released from red cells when they die, either naturally or after infections such as malaria. This protein exists in two forms, 1 or 2; the possible combinations of the protein are 1–1, 1–2, or 2–2. 2–2 is the least efficient at binding to the free haemoglobin. Previous work has suggested that the haptoglobin type people carry may affect how they respond to malaria, and whether they become iron deficient. However, it is not known whether the haptoglobin type of children will affect their risk of being anaemic in areas where malaria is very common.

**What Did the Researchers Do and Find?**

A total of 780 rural Gambian children between 2 and 6 years of age were examined at the start and end of the malaria season. The authors found that average haemoglobin levels fell over the malaria season, and children who had the haptoglobin 2–2 type had the greatest drop compared to other children. When the authors looked at a number of other factors, including the children's age, haemoglobin at the start of the season, and which village they were from, the haptoglobin type still predicted whether haemoglobin levels would fall at the end of the malaria season.

**What Do These Findings Mean?**

These findings suggest that the haptoglobin type a child has may have be an important influence on whether that child gets anaemia in areas where malaria is very common. It is not clear why this haptoglobin type is frequent in this population if it has an adverse effect on haemoglobin levels; one possibility is that the 2–2 type may protect against life-threatening malaria. Further research needs to be done to check this finding and to investigate exactly the potential mechanisms of anaemia.

**Where Can I Get More Information Online?**

The World Health Organization has a page of information on anaemia:

http://www.who.int/topics/anaemia/en

Medline Plus has a page of links to malaria:

http://www.nlm.nih.gov/medlineplus/malaria.html

and an explanation of what haptoglobin is:

http://www.nlm.nih.gov/medlineplus/ency/article/003634.htm

## Abbreviations

CI _95%_: 95% confidence interval
G6PD: glucose-6-phosphate
Hb: haemoglobin
Hp: haptoglobin
LDL: low-density lipoprotein
