# Supplementary material for: Seasonal Childhood Anaemia in West Africa Is Associated with the Haptoglobin 2-2 Genotype
Source: PLoS Med. 2006 May 2;3(5):e172. doi: 10.1371/journal.pmed.0030172 (PMC1450017; doi:10.1371/journal.pmed.0030172)
Supplement: Supplementary file 1 [file pmed.0030172.st001.doc]

**Supplementary Information**

**Table S1.** Oligonucleotide Sequences of the Haptoglobin Primers.

**Primer Nucleotide Sequences (5’-3’)**

F3   CAGGAGTATACACCTTAAATG

S2   TTATCCACTGCTTCTCATTG

C42   TTACACTGGTAGCGAACCGA

C72   AATTTAAAATTGGCATTTCGCC

C51   GCAATGATGTCACGGATATC

The forward and reverse primers are from Yano et al^17^.  F3 and C42 are primers for the Hp 2 reaction, C51 and S2 for the Hp1S reaction and F3 and C72 for the Hp1F reaction.
