# Supplementary material for: Seasonal Childhood Anaemia in West Africa Is Associated with the Haptoglobin 2-2 Genotype
Source: PLoS Med. 2006 May 2;3(5):e172. doi: 10.1371/journal.pmed.0030172 (PMC1450017; doi:10.1371/journal.pmed.0030172)
Supplement: Supplementary file 2 [file pmed.0030172.st002.doc]

**Table S2.** Allele Specific Polymerase Chain Reaction Conditions

|  | Hp2 reaction (µl) |  | Hp1S reaction (µl) |  | Hp1F reaction (µl) |
| --- | --- | --- | --- | --- | --- |
| PE buffer II 10x | 1.5 |  | 1.5 |  | 1.5 |
| MgCl2 25 mM | 1.5 |  | 1.5 |  | 1.2 |
| dNTPs 8 mmol total | 0.6 |  | 0.6 |  | 0.6 |
| Hp (F/R) primer mix  5µM (each) | F3+C42  0.72 |  | C51+S2  0.72 |  | F3+C72  0.72 |
| Control primer mix CCR6 1.25µM (each) | (F+R)  0.36 |  | (F+R)  0.36 |  | (F+R)  0.36 |
| TAQ GOLD 5u/µL | 0.12 |  | 0.12 |  | 0.12 |
| H2O (milliQ 18.2 *) | 8.7 |  | 8.7 |  | 9.0 |
| Volume of Mix | 13.5 |  | 13.5 |  | 13.5 |
| DNA (10ng/µl) | 1.5 |  | 1.5 |  | 1.5 |
| Total Volume | 15 |  | 15 |  | 15 |
| PCR cycling conditions | 94ºC 14 min  94ºC 40 sec\|  55.8ºC 1 min \| 38 cycles  72ºC 2 min \|  72ºC 7 min |  | 94ºC 14 min  94ºC 40 sec\|  59 ºC 1 min \| 38cycles  72ºC 2 min \|  72ºC 7 min |  | 94ºC 14 min  94ºC 40 sec\|  55.6ºC 1 min \| 38 cycles  72ºC 1 min \|  72ºC 5 min |

 The Hp1F reaction was optimized by reducing the amount of MgCl_2,_ doubling the amount of haptoglobin primer mix, and changing the annealing temperature to 55.6ºC.
