# Supplementary material for: Seasonal Childhood Anaemia in West Africa Is Associated with the Haptoglobin 2-2 Genotype
Source: PLoS Med. 2006 May 2;3(5):e172. doi: 10.1371/journal.pmed.0030172 (PMC1450017; doi:10.1371/journal.pmed.0030172)
Supplement: Supplementary file 3 [file pmed.0030172.st003.doc]

**Table S3.** Oligonucleotide Sequences of First Round and Extension Primers Designed by Sequenom Primer Designer Software™ for G6PD (A and A-) and HbS.

| **Genotype** |  | **First round primers** |  | **Extension primers** |
| --- | --- | --- | --- | --- |
| **G6PD A-** |  | ACGTTGGATGATGGCCTTCTGCCCGAAAAC  ACGTTGGATGACTCTGTTTGCGGATGTCAG |  | CTGCCCGAAAACACCTTCATC |
|  |  |  |  |  |
|  |  |  |  |  |
| **G6PD A** |  | ACGTTGGATGATGATGCAGCCTCCTACCAG  ACGTTGGATGTAGAAGAGGCGGTTGGCCTG |  | AGCGCCTCAACAGCCACATG |
|  |  |  |  |  |
|  |  |  |  |  |
| **HbS** |  | ACGTTGGATGTTCATCCACGTTCACCTTGC  ACGTTGGATGAAACAGACACCATGGTGCAC |  | AGGGCAGTAACGGCAGACTTCTCC |
|  |  |  |  |  |
